# 介绍一种骨髓活检组织脱钙方法

**DOI:** 10.3760/cma.j.cn121090-2023915-00121

**Published:** 2024-04

**Authors:** 先稳 彭, 霂 缐, 宁溥 班, 琦 孙

**Affiliations:** 1 中国医学科学院血液病医院（中国医学科学院血液学研究所），血液与健康全国重点实验室，国家血液系统疾病临床医学研究中心，细胞生态海河实验室，天津 300020 State Key Laboratory of Experimental Hematology, National Clinical Research Center for Blood Diseases, Haihe Laboratory of Cell Ecosystem, Institute of Hematology & Blood Diseases Hospital, Chinese Academy of Medical Sciences & Peking Union Medical College, Tianjin 300020, China; 2 天津医学健康研究院，天津 301600 Tianjin Institutes of Health Science, Tianjin 301600, China

## Abstract

骨髓活检是血液病理诊断的重要手段之一，对于多种良、恶性淋巴造血系统疾病均具有决定性的诊断意义，其诊断价值包括形态学观察，以及免疫组化、遗传学和分子生物学等辅助检测。由于骨髓活检组织本身的特殊性，脱钙是前期处理过程中必不可少的环节，其目的是将骨组织中钙盐去掉，保留完整的胶原纤维成分，以利于组织切片以及避免染色时掉片。如果骨髓活检脱钙不充分，则难以制片，但如果脱钙过度，又会严重破坏组织抗原活性，因此，一种脱钙效率高且抗原破坏小的脱钙方法对于临床病理骨髓活检十分重要。本文介绍一种高效且抗原破坏较小的骨髓活检组织脱钙方法：12％甲酸−8％盐酸脱钙液室温摇床脱钙，供广大同仁参考和指正。

## Abstract (en)

Bone marrow biopsy is one of the important means of hematopathological diagnosis, which has decisive diagnostic significance for various benign and malignant lymphohematopoietic system diseases. Its diagnostic value includes morphological observation, immunohistochemistry, genetics, and molecular biology testing. Owing to the unique nature of bone marrow biopsy, decalcification is an essential step in the pre-treatment process. Its purpose is to remove calcium from bone tissue, preserve intact collagen fiber components, facilitate tissue sectioning, and prevent tissue detachment during staining. If bone marrow biopsy lacks sufficient decalcification, preparing a section is difficult. Conversely, if decalcification is excessive, it can seriously disrupt tissue antigen activity. Therefore, a decalcification method with high decalcification efficiency and mild antigen damage is essential for bone marrow biopsy. This article introduces a bone marrow biopsy tissue decalcification method with high efficiency and less antigen loss: decalcification is performed at room temperature with 12％ formic acid and 8％ hydrochloric acid decalcification solution on a shaker.

骨髓活检是血液病理诊断的重要方法之一，而制备良好的骨髓活检切片则是诊断正确的重要前提。骨髓活检组织由无机矿物质、有机基质、水及细胞成分构成，其中的无机矿物质主要为羟基磷灰石，也称碱式磷酸钙［Ca_10_（PO_4_）_6_（OH）_2_］，其沿基质胶原纤维的轴线排列并加强胶原基质，以保证骨组织的硬度和坚固性[1]，但同时导致骨髓活检切片时无法得到连续完整的组织切面，因此，磷酸钙是影响骨髓组织切片质量的主要因素。对骨髓组织进行脱钙处理就是通过去除磷酸钙来软化骨髓组织的过程，是骨髓活检标本前期处理过程中必不可少的步骤，然而，骨髓组织脱钙面临的最大问题是如何快速脱钙，同时避免组织的抗原、核酸等成分破坏，以最大程度满足病理形态观察、免疫组化以及分子生物学等辅助检测的需求。基于此，本文介绍一种笔者所在实验室使用的骨髓活检组织脱钙方法，供广大同仁参考和指正。

一、国内骨髓活检组织脱钙的发展历程

国际上关于骨髓活检技术的文献报道可追溯到20世纪50年代，我国稍晚。1977年，中国人民解放军第一四五医院病理科周瑞成发表文章《骨组织的快速脱钙》[2]，文章中使用的脱钙液为盐酸-冰醋酸-甲醛脱钙液，其配制方法为：甲醛50 ml、冰醋酸25 ml，加蒸馏水至500 ml为甲液；盐酸40 ml加蒸馏水至500 ml为乙液，将甲液与乙液1:1混合即可；脱钙时加热至50～60 °C，松质骨使用此脱钙液只需5～20 min即可完成脱钙，但该方法的缺点是脱钙温度高，具有导致组织肿胀和消化的风险，而且酸和醛在此温度下会挥发，易造成空气污染。1985年，上海第二医学院杜心垿等[3]提到实验室自1978年开始使用“蚁酸和蚁醛混合液”脱钙，此脱钙液脱钙会影响铁染色结果。1995年，北京中日友好医院病理科王颖等[4]使用的脱钙液为3％盐酸−8％甲酸混合液。

本单位前身为1957年中央卫生部与中国人民解放军总后勤部卫生部联合组建的输血及血液学研究所，是我国第一个基础与临床结合的综合性血液学科学研究单位，也是我国第一家血液系统疾病专科医院[5]。骨髓病理作为血液系统疾病必不可少的诊断方法之一，对各类淋巴造血系统疾病的确诊与鉴别具有非常重要甚至决定性意义，因此，骨髓活检也成为本单位病理科的主要诊断项目和特色诊断项目，然而，骨髓活检的组织处理具有一定挑战性，主要原因是骨髓组织具有“骨”这一特性，因此，组织前期处理过程中需要脱钙。在20世纪80年代，本单位实验室的陈文杰和陈辉树两位教授根据多年从事骨髓病理诊断研究工作的经验，采用2％硝酸溶液（浓硝酸2 ml，蒸馏水98 ml）进行骨髓活检脱钙，脱钙时间为2 h[6]；之后，陈辉树教授等通过不断实践，发现5％硝酸脱钙2～2.5 h比2％硝酸脱钙2～2.5 h更彻底，两者对抗原的破坏亦无明显差别，因此在20世纪90年代，实验室常规采用5％硝酸用于骨髓活检脱钙[7]，其配制方法为：浓硝酸5 ml加入95 ml蒸馏水即可，蒸馏水可用4％甲醛液替代。实验室也曾使用过兼具固定及脱钙作用的Bouin液（饱和苦味酸液85 ml、甲醛10 ml、乙酸5 ml，混合即可）及EDTA脱钙液（用100 ml pH 6.8～7.0的PBS溶解10 g EDTA即可），使用Bouin液对骨髓活检组织脱钙，适用于形态学观察，但不适合进行免疫组化染色；EDTA脱钙液对骨髓抗原活性保存效果最好，适用于免疫组化染色及分子生物学检测，但唯一的缺点是脱钙时间长，无法满足本单位对骨髓病理报告周期的要求。

二、12％甲酸−8％盐酸脱钙液的临床应用

为了能兼顾脱钙效果、后续检测的适用性以及骨髓病理报告周期等，本实验室持续探索能充分且快速脱钙、并减少抗原损害的脱钙液，在2000年实验室开始探索混合酸脱钙液，混合酸脱钙液主要为无机酸与有机酸混合，该脱钙液结合了无机酸脱钙速度快、有机酸对组织损伤小的特点，相对于单用无机酸脱钙液或有机酸脱钙液，混合酸脱钙液在脱钙速度、抗原损害、苏木精-伊红（HE）染色效果等方面综合性能最优，通过不断的实验验证，发现12％甲酸−8％盐酸脱钙液不仅能够高效脱钙，而且抗原损害程度与5％硝酸脱钙相比显著降低，因此，12％甲酸−8％盐酸脱钙液成为本实验室首选的脱钙液，其配制方法为800 ml蒸馏水中加入120 ml甲酸（HCOOH, w/％≥88.0）及80 ml盐酸（HCl, w/％为36.0～38.0），使用此脱钙液再联合摇床震荡，在室温下只需约2 h即可完成骨髓组织脱钙，而且所制切片切面完整、无刀痕、厚薄均匀一致，HE染色显示骨髓细胞形态和结构全面清晰，细胞核、细胞质颜色对比鲜明，网状纤维染色清晰、背景干净易评估，免疫组化染色显示细胞核抗原、细胞质抗原及细胞膜抗原均表达良好，阳性信号定位准确[8]。使用该脱钙液对骨髓活检组织进行脱钙不仅经济高效而且抗原损害小，此方法沿用至今。经过12％甲酸−8％盐酸脱钙液处理后骨髓活检组织的HE染色、Gomori染色、PAS染色及常用血液系统疾病免疫组化检测指标染色结果见图1、2。

**图1:**
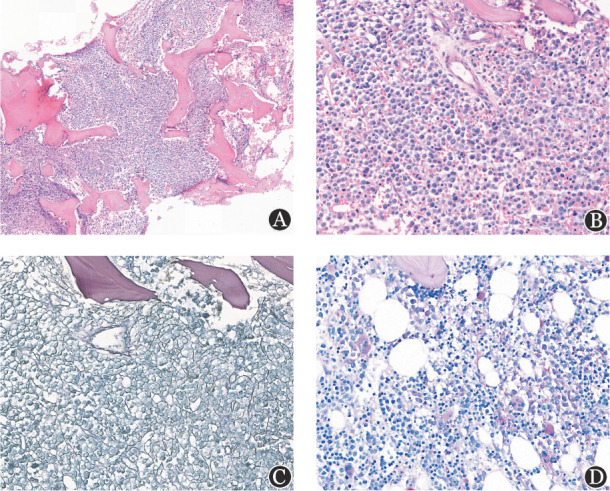
使用12％甲酸−8％盐酸脱钙液脱钙的骨髓活检染色结果 **注** **A** HE染色显示组织切面完整、无刀痕（×100）；**B** HE染色示细胞结构清晰，细胞质与细胞核红蓝染色对比鲜明（×400）；**C** Gomori染色显示骨髓活检内小血管周围网状纤维着色（为阳性内对照），造血细胞区域网状纤维纤细、状如发丝，背景干净、清晰，无非特异性着色（×400）；**D** PAS染色示粒系细胞、巨核细胞胞质阳性（呈玫红色），红系细胞胞质阴性，阴、阳性反应对比鲜明（×400）

**图2:**
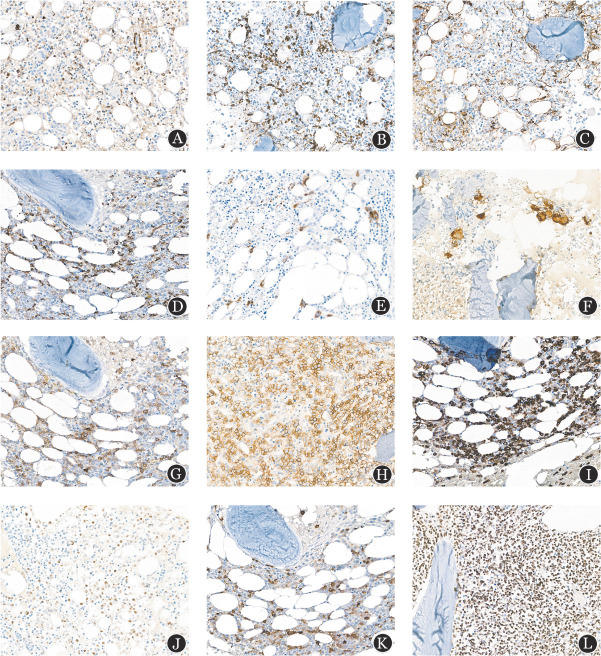
使用12%甲酸−8%盐酸脱钙液脱钙的骨髓活检免疫组化染色（阳性信号定位准确、清晰，背景干净，无非特异性着色，×400） **注** **A** BCL2染色；**B** CD3染色；**C** CD10染色；**D** CD34染色；**E** CD42b染色；**F** CD61染色；**G** CD117染色；**H** E-cadherin染色；**I** MPO染色；**J** c-Myc染色；**K** Lysozyme染色；**L** PAX5染色

三、12％甲酸−8％盐酸脱钙液的缺点

与其他酸类脱钙液相同，12％甲酸−8％盐酸脱钙液同样具有“酸”的性质，因此对核酸具有一定程度的破坏作用[9]，脱钙后的骨髓组织进行分子生物学检测，结果欠佳。本实验室曾选取使用新鲜骨髓液进行基因重排检测结果为阳性的病例，再使用同时钻取的骨髓活检组织进行基因重排检测，结果部分标本核酸提取质量不佳，部分标本即使提取到有效的DNA，但检测结果为假阴性，仅少数病例骨髓活检的基因重排结果与骨髓液一样为阳性，可见该脱钙液明显影响分子生物学检测结果的准确性。尽管EBER原位杂交检测结果阳性率略高，阳性定位正确（图3），但仍然存在假阴性的问题，因此，我们不建议使用经12％甲酸−8％盐酸脱钙液处理的骨髓活检标本进行分子生物学检测。如果临床需要，建议首选新鲜骨髓液检测，或者对骨髓活检组织使用EDTA脱钙处理，以确保检测结果的准确性。

**图3:**
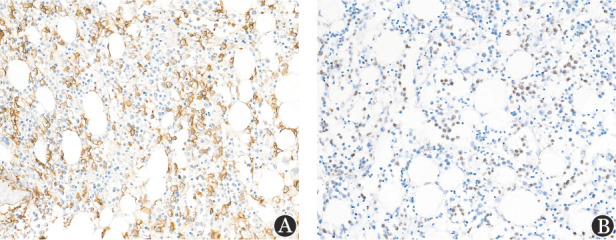
使用12％甲酸−8％盐酸脱钙液脱钙的结外NK/T细胞淋巴瘤累及骨髓患者的骨髓活检组织CD56免疫组化染色及EBER原位杂交染色结果（×400） **注A** CD56染色；**B** EBER染色

四、结语

目前，国内关于骨髓活检组织的脱钙方法缺乏指导性规范，各医院病理科采用的脱钙方法不尽相同，本文结合实践经验及查阅相关文献认为：①单纯的有机酸脱钙液脱钙速度慢且不彻底。②单纯的无机酸脱钙液脱钙效率高，但是组织抗原破坏严重。③EDTA脱钙液最温和，适合免疫组化染色及分子生物学等研究，但是脱钙时间过长，不适合常规临床病理诊断使用。④我们通过实践证明，12％甲酸−8％盐酸脱钙液兼有脱钙时间短（2 h），脱钙后对骨髓组织切片HE染色、特殊染色及免疫组化染色影响小的优点，能够满足临床病理日常大量骨髓标本有效脱钙的需求，如配合摇床震荡使用，能提高脱钙效率、保证脱钙质量，而且经济方便，是我们推荐的一种脱钙方法；但该方法同样存在破坏核酸的问题，因此不适合用于分子生物学检测，存在假阴性的风险。希望本文对未来我国骨髓活检组织脱钙流程的标准化、规范化以及相关指南共识的制定提供一定的参考依据。
